# Difference in the prevalence of non-fatal suicidal behaviours in patients with unipolar and bipolar depression in China: a meta-analysis

**DOI:** 10.18632/oncotarget.24554

**Published:** 2018-02-22

**Authors:** Peiwei Shan, Liuzhen Hu, Dali Xu, Chunxia Fang, Jie Li, Deguo Jiang, Wei Zhang, Chuanjun Zhuo

**Affiliations:** ^1^ The Department of Psychiatry, Wenzhou Seventh People's Hospital, Wenzhou City, 325000, Zhejiang Province, China; ^2^ The Department of Psychiatry, Changzhou Dean Hospital, Changzhou City, 213000, Jiangsu Province, China; ^3^ The Department of Psychiatry, Tianjin Anding Hospital, Tianjin Mental Health Center, Tianjin City, 300222, China

**Keywords:** unipolar depression, bipolar depression, non-fatal suicidal behaviours

## Abstract

Difference in the prevalence of non-fatal suicidal behaviours in patients with unipolar and bipolar depression in China is rarely reported. We conducted a meta-analysis to examine this difference. Major Chinese and English literature databases were searched online to collect studies comparing the prevalence of non-fatal suicidal behaviours of patients with unipolar and bipolar depression. The quality of the included studies was assessed according to the matching principle. Risk difference (RD) of prevalence rates of non-fatal suicidal behaviours between patients with unipolar and bipolar depression was calculated. A total of 16 studies, containing 1678 cases with unipolar depression and 1069 cases with bipolar depression, were included. Differences in rates of suicidal ideation and attempt were not statistically significant between patients with unipolar and bipolar depression, but rates of overall non-fatal suicidal behaviours in patients with unipolar depression was significantly higher than those with bipolar depression (RD: 0.28, 95% CI: 0.20, 0.36). In summary, the rate of overall non-fatal suicidal behaviours of patients with unipolar depression is higher than that of patients with bipolar depression in China.

## INTRODUCTION

Because most bipolar disorder patients were first manifested with depression, at least 40% of bipolar disorder patients were misdiagnosed as unipolar depression, and at least 20% of patients with major depressive disorder who had received antidepressant treatment also changed to bipolar disorder in a subsequent diagnosis [1–4]. The clinical treatment of bipolar depression is significantly different from that of unipolar depression; therefore, the correct diagnosis of bipolar disorder is a prerequisite for an appropriate treatment. However, patients with depression typically cannot provide a clear history of manic or hypomanic episode; thus, the early prediction of bipolar disorder based on the characteristics of the patients’ first depressive episode may be one of the important ways of reducing missed diagnosis and misdiagnosis. Accordingly, some researchers have proposed the concept of “soft bipolarity” [5], which advocates predicting future manic or hypomanic episodes based on the current clinical characteristics of patients with depression. Researchers in China and other countries have identified a number of risk factors that may suggest soft bipolarity by comparing the clinical characteristics of unipolar depression and bipolar depression, such as early age at onset, cyclothymic personality, family history of bipolar depression, and suicidal behaviour [6–8]. It is generally believed that suicidal ideation and attempt are more common in patients with bipolar depression than in patients with unipolar depression. Therefore, suicidal behaviour is also recommended as one of the predictive signs for unipolar and bipolar depression [8]. However, findings from the comparative analyses of the clinical characteristics of Chinese patients with unipolar and bipolar depression are conflicting. For example, Zhang et al. [9] found that, although the number of previous suicidal attempts in patients with unipolar depression is less than those with bipolar depression, the difference in rates of attempted suicide was not significant. Wang et al. [10] found that the score of suicide subscale on the Hamilton Depression Scale of patients with unipolar depression was significantly lower than that of patients with bipolar depression. It is also worth noting that the majority of these prior comparative studies on the suicide risk of patients with unipolar and bipolar depression in China are small-scale studies, leading to insufficient statistical power to detect the true difference between the two groups of patients. Therefore, to provide the evidence for the clinical prediction of bipolar disorder in patients with depression, it is necessary to summarize findings from existing empirical studies by using meta-analysis.

## RESULTS

### Results of literature search and characteristics of included studies

A total of 18 studies comparing suicidal behaviours between Chinese patients with unipolar and bipolar depression, were retrieved; two [9, 10] were excluded due to unavailable data on the numbers of patients with the specific suicidal behaviours. Finally 16 studies [13–28] were included in this meta-analysis, containing 1678 cases with unipolar depression and 1069 with bipolar depression. Of the 16 studies, five [13, 14, 21, 26, 28] reported rate of suicidal ideation, five [13, 14, 18, 26, 28] reported suicide attempt, and nine [16, 17, 19, 20, 22–25, 27] reported total suicidal behaviours (including ideation, plan, and attempt). All patients with bipolar depression of included studies had depressive episode. The general information of the included studies is presented in Table 1.

**Table 1:** The general information of the studies included in the meta-analysis

Study	Unipolar depression				Bipolar depression				Matched gender and age^*^
	Number of cases	Suicidal ideation	Suicidal attempt	Suicidal behaviour	Number of cases	Suicidal ideation	Suicidal attempt	Suicidal behaviour	
Xiang Tianwei, 1983	22	3	15	--	7	0	3	--	Unknown
Chen Bixia, 1991	58	46	33	--	71	32	22	--	Unknown
Dong Xinyong, 2000	32	--	21	--	60	--	18	--	Unknown
Li Yiyun, 2000	32	--	--	21	40	--	--	10	Unknown
Jin Weidong, 2004	55	--	--	44	68	--	--	48	Unmatched
Qi Shuguang, 2005	115	--	59	--	184	--	31	--	Unmatched
Feng Zheng, 2006	35	--	--	20	32	--	--	8	Unmatched
Gao Yuanjun, 2007	31	--	--	13	28	--	--	5	Matched
Yang Jianhong, 2008	144	72	--	--	96	38	--	--	Matched
Wu Sheng, 2009	48	--	--	32	35	--	--	10	Unmatched
Hu Weisheng, 2009	25	--	--	21	68	--	--	36	Unknown
Wu Xiaohe, 2010	69	--	--	51	98	--	--	34	Unmatched
Guo Yahui, 2012	100	--	--	45	47	--	--	15	Unmatched
Ye Kaiwen, 2014	56	37	32	--	53	48	41	--	Matched
Li Geng, 2015	148	--	--	89	57	--	--	16	Unknown
Wu Zhiguo, 2015	708	422	81	--	125	77	29	--	Matched

*Some studies did not report the gender and age composition of the patients with unipolar and bipolar depression. Whether the matching principal was satisfied could not be determined, therefore the methodological quality of such studies was recorded as “unknown”.

### Methodological quality of included studies

The numbers of studies with methodological quality being rated as “high”, “low”, and “unknown” were four, six, and six, respectively, as shown in Table 1.

### Difference in the rates of suicidal ideation between patients with unipolar and bipolar depression

**Figure 1:**
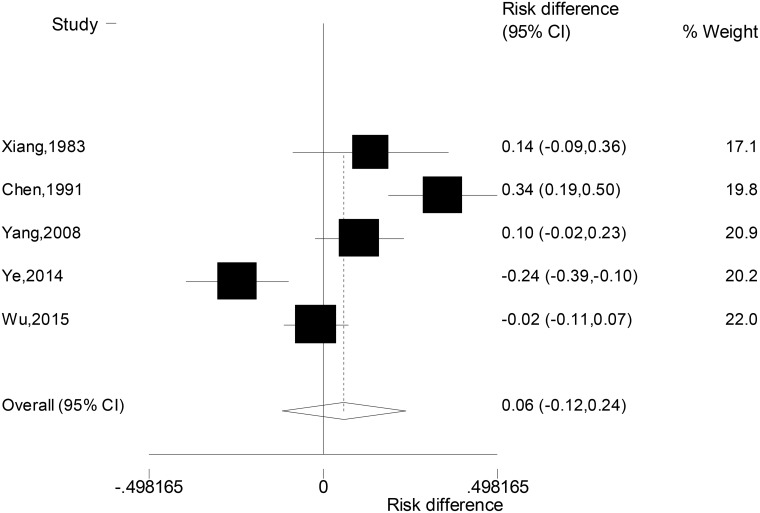
Forest plot for the difference in the suicidal ideation rates between patients with unipolar and bipolar depression

Due to the significant heterogeneity across the five studies (c^2^ = 32.19, *P* < 0.001), random-effect model was used to combine the differences in the rates of suicidal ideation between patients with unipolar and bipolar depression. Results (Figure 1) showed that the suicidal ideation rates of the two groups of patients were not significantly different (RD: 0.06, 95% CI: –0.12, 0.24).

### Difference in the rate of suicidal attempts between patients with unipolar and bipolar depression

**Figure 2:**
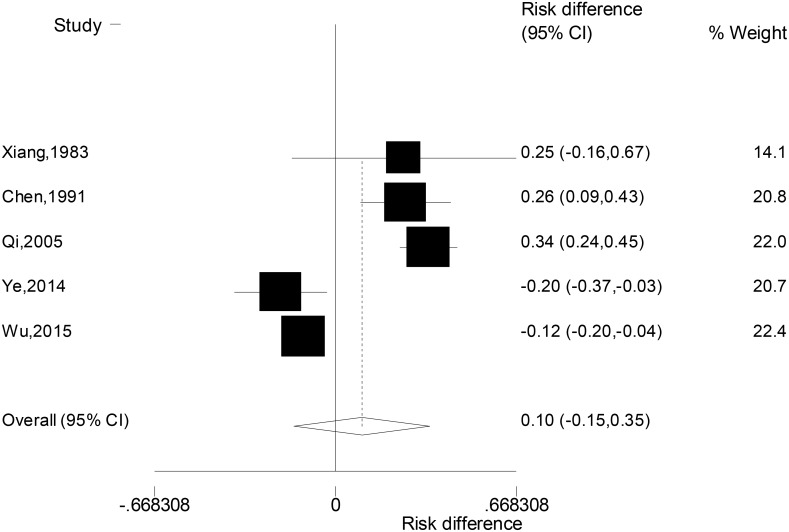
Forest plot for the difference in the rates of suicide attempts between patients with unipolar and bipolar depression

Due to the significant heterogeneity across five studies (c^2^ = 62.7, *P* < 0.001), random-effect model was used to combine the differences in the rates of suicidal attempt between patients with unipolar and bipolar depression. Results (Figure 2) showed that the suicide attempt rates of the two groups of patients were not significantly different (RD: 0.10, 95% CI: –0.15, 0.35).

### Difference in the rate of suicidal behaviour between patients with unipolar and bipolar depression

**Figure 3:**
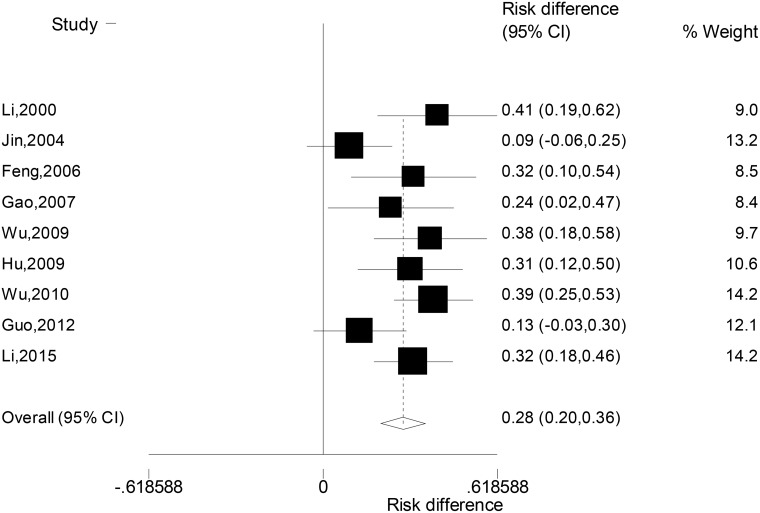
Forest plot for the difference in the rates of suicidal behaviour between patients with unipolar or bipolar depression

Due to the significant heterogeneity across nine studies (c^2^ = 14.4, *P* = 0.072), random-effect model was used to combine the differences in the rate of suicidal behaviour between patients with unipolar and bipolar depression. Results (Figure 3) showed that the suicidal behaviour rates of the two groups of patients were significantly different, with higher rates in unipolar than bipolar depression (RD: 0.28, 95% CI: 0.20, 0.36).

## DISCUSSION

Unipolar and bipolar depression are different disease entities. Unlike unipolar depressive disorder, the clinical manifestations and treatment of bipolar depression is more complex, for example, patients with bipolar depression have a worse prognosis [8, 29]. Fang et al. [8] argued that patients with bipolar depression have a high rate of non-fatal suicidal behaviour (suicidal ideation and attempt) and a low rate of completed suicide whereas patients with unipolar depression have a low rate of non-fatal suicidal behaviour and a high rate of completed suicide; thus, non-fatal suicidal behaviour can be used as a differential factor for the differentiation between unipolar and bipolar depression.

Findings from this meta-analysis show that there was no significant difference in the rates of suicidal ideation and attempt between patients with unipolar and bipolar depression; however, the prevalence of overall nonfatal suicidal behaviours in patients with unipolar depression was significantly higher than that in patients with bipolar depression (by 28% on average). The insignificant difference in a specific non-fatal suicidal behaviour between the two groups may be related to the small number of included studies, with insufficient statistical power. However, the significant difference in the prevalence of total non-fatal suicidal behaviours contradicts the traditional perception of the high suicide risk in patients with bipolar depression relative to unipolar depression, suggesting that using non-fatal suicidal behaviour to predict future manic or hypomanic episodes in patients with depression is still questionable.

Depression is a well-recognized strong risk factor for suicidal behaviours (from suicidal ideation to suicide death), and the risk of suicidal behaviour is also strongly associated with the depressive symptoms of these patients [30]. A previous comparison of the clinical characteristics in patients with unipolar and bipolar depressive episodes have shown that significantly higher prevalence of non-fatal suicidal behaviours in the patients with unipolar depressive episodes than that in patients with bipolar depressive episodes, even though the severity of depression between the two groups was comparable [16]. Thus, the difference in the severity of depression between the two groups of patients cannot explain the difference in the non-fatal suicidal behaviours. The difference in the incidence of suicidal behaviours in patients with unipolar and bipolar depressive episodes may be related to other characteristics associated with the disease; for example, depression in patients with unipolar depression may be more accompanied by melancholic features than in patients with bipolar depression.

### Limitations

There are several limitations in this meta-analysis. First, the methodological quality of most included studies was poor or “unknown”. Second, type I or type II bipolar depressive episodes were not differentiated in the bipolar depressive episodes in the original included studies, but the risk of suicide in patients with different types of bipolar disorders should be different [31]. Therefore, the results of this study could not rule out the confounding effect of the type of bipolar disorders. Third, detailed information on the assessments of non-fatal suicidal behaviours were not unavailable from included studies because these studies focused on many clinical characteristics. Despite these limitations, this study reveals the lower risk of non-fatal suicidal behaviours in patients with bipolar depression compared to unipolar depression, suggesting that suicidal behaviour should not be used as a clinical indicator in predicting the likelihood of depressive episodes in patients with bipolar disorder.

## MATERIALS AND METHODS

### Inclusion and exclusion criteria

The inclusion criteria were as follows: 1) published Chinese and English studies, which were designed to cross-sectionally compare the clinical characteristics of Chinese patients with unipolar and bipolar depression and had reported the rates of non-fatal suicidal behaviours (death desire, suicidal ideation, plan, attempt) of the two groups of patients; 2) the diagnoses of patients with unipolar and bipolar depression must be made based on widely used diagnostic criteria for mental disorders, irrespective of the diagnostic criteria used; and 3) the study subjects were adults. The exclusion criteria were as follows: 1) the study did not provide specific data on cases with suicidal behaviours; and 2) reviews and duplicated publications.

### Search strategy

We searched Chinese and English literature databases online (up to 13 August 2017), including the China National Knowledge Infrastructure (CNKI), Wanfang, CQVIP, PubMed, Embase, and PsycINFO. The keywords for the search were “mood or affective disorder”, “bipolar”, “mania”, “depress*”, “clinical characteristics or features”, and “suicid*”.

### The screening of eligible studies and data extraction

After reading the titles and abstracts, the studies meeting the inclusion criteria were included, and duplicate publications were excluded. The data extracted from these included studies were first author, publication date, gender composition (% of males) and mean age of patients with unipolar and bipolar depression, and types of reported non-fatal suicidal behaviour, as well as their corresponding numbers of cases. Literature search, screening, data extraction, and quality assessment of eligible studies were independently completed by the first and third authors of this study. Collected data were cross-checked and the inconsistencies were resolved through discussion. If necessary, the second author was invited to solve any disagreement.

### Quality assessment of the included studies

According to previous studies [11, 12], the methodological quality assessment of included studies focused on the matching of gender and age compositions of the two groups of patients. If the two variables were comparable (matched), this individual study was considered as having high quality.

### Statistical methods

The meta-analysis was performed using the meta-analysis module of Stata 12.0 software. Heterogeneity was analysed using the c^2^ test, with *P* ≥ 0.10 suggesting no significant heterogeneity between the studies, and a fixed-effect model was used for pooling; otherwise, a random-effect model was used for the pooling. The combined effect measure was risk difference (RD), that is, the difference in the rates of suicidal behaviour in patients with unipolar and bipolar depression. The significance level of the test was two-sided *P* < 0.05.

## CONCLUSIONS

In summary, the rate of overall non-fatal suicidal behaviours of patients with unipolar depression is higher than that of patients with bipolar depression in China.

## References

[1] Hu C, Xiang YT, Ungvari GS, Dickerson FB, Kilbourne AM, Si TM, Fang YR, Lu Z, Yang HC, Chiu HF, Lai KY, Hu J, Chen ZY (2012). Undiagnosed bipolar disorder in patients treated for major depression in China. J Affect Disord.

[2] Perlis RH (2005). Misdiagnosis of bipolar disorder. Am J Manag Care.

[3] Vöhringer PA, Perlis RH (2016). Discriminating Between Bipolar Disorder and Major Depressive Disorder. Psychiatr Clin North Am.

[4] Agius M, Murphy CL, Zaman R (2010). Under-diagnosis of bipolar affective disorder in A bedford CMHT. Psychiatr Danub.

[5] Kuppili PP, Yadav P, Pattanayak RD (2017). Concept and Identification of “Soft Bipolarity” in Patients presenting with Depression: Need for Careful Screening by Physicians. J Assoc Physicians India.

[6] Takeshima M, Oka T (2013). A comprehensive analysis of features that suggest bipolarity in patients with a major depressive episode: which is the best combination to predict soft bipolarity diagnosis?. J Affect Disord.

[7] Jin W, Chen J, Xing B, Chen Z, Liu J, Mao F, Xu L, Wang H, Tang X, Chen Z (2007). [Establishment and evaluation the advising criteria of soft bipolar disorder]. [Article in Chinese]. Chinese Journal of Behavioral Medicine and Brain Science.

[8] Fang Y, Wang Z (2011). [Current status and future trends in clinical research of bipolar disorder]. [Article in Chinese]. Shanghai Jingshen Yixue.

[9] Zhang M (2008). Comparison study about the cognitive ability between unipolar and bipolar disorder depression.

[10] Wang P, Yang C, Lian Z (2014). A comparative study of clinical data and characteristics of unipolar depression and bipolar depression. Chinese Journal of General Practice.

[11] Xu Y, Zhong B, Cao X, Li B, Deng F, Liu X, Liu T (2014). Effects of smoking on numbers of hospital admissions and length of of hospital study among Chinese patients with schizophrenia-a meta analysis. Chin J Drug Depend.

[12] Xu Y, Zhong B, Li Y, Cao X, Liu X, Li B (2017). Rate of smoking before the first psychotic episode among Chinese schizophrenia smokers: a meta-analysis. Sichuan Mental Health.

[13] Tian W, Zheng H (1983). The characters of 52 depression patients. Chinese Journal of Nervous and Mental Diseases.

[14] Chen B, Xu J (1991). [Bipolar and the unipolar depression]. [Article in Chinese]. Shanghai Jingshen Yixue.

[15] Dong X, Xu W, Lin Z, Yaoming LV (2000). Comparison the clinical features of bipolar and unipolar depression. J Clin Psychiatry.

[16] Li Y, Chen D, Ji J (2000). Comparison the clinical features of bipolar and unipolar depression. J Clin Psychiatry.

[17] Jion W, Chen H, Xing B, Wang H, Tong Z, Feng B, Xu L, Li N, Wang Y (2004). Clinical analysis of 68 cases of bipolar disorder with first-episode depression. J Clin Psychiatry.

[18] Qi S, An B, Zhang Y, Dong X (2005). A study on the genetic effects of suicidal behavior in unipolar depression and bipolar depression. Chinese Journal Of Nervous And Mental Diseases.

[19] Feng Z, Liao C, Xu Y, Gao H (2006). Comparison the clinical features of bipolar and unipolar depression. Medical Journal of Chinese People's Health.

[20] Gao Y (2007). Analysis of the clinical features of bipolar and unipolar depression. Med Infant.

[21] Yang J, Shen X, Li L, Lan G (2008). [Comparison the clinical features of bipolar and unipolar depression]. [Article in Chinese]. Shanghai Jingshen Yixue.

[22] Wu S (2009). Analysis of the clinical characters of bipolar disorder. Chongqing Medicine Journal.

[23] Hu W (2009). Clinical analysis of 93 cases of affective disorder. China Practical Medical.

[24] Wu X, Zhang C (2010). A comparative study of clinical features of bipolar disorder and unipolar depression. Medical Journal of Chinese People's Health.

[25] Guo Y, Ma Z, Wang Y (2012). A comparative study of clinical features of unipolar depression and bipolar depression. Chinese Journal of Practical Medicine.

[26] Ye K, Zhang S (2014). Analyses of clinical features of unipolar and bipolar disorder patients with psychotic symptoms. Journal of Clinical Psychosomatic Diseases.

[27] Li G, Cao Y, Yong S (2015). A comparative study of clinical features of unipolar depression and bipolar depression episode. Journal of Ningxia Medical University.

[28] Wu Z, Cao L, Li H, Qiu M, Li N, Xiang H, Huang Y, Liao L (2015). Psychiatric comorbidities in patients with major depressive disorder and patients with bipolar II depression in a psychiatric setting. Chinese Journal of Psychiatry.

[29] das Neves Peixoto FS, de Sousa DF, Luz DC, Vieira NB, Gonçalves Júnior J, Dos Santos GC, da Silva FC, Rolim Neto ML (2017). Bipolarity and suicidal ideation in children and adolescents: a systematic review with meta-analysis. Ann Gen Psychiatry.

[30] Dong M, Wang SB, Li Y, Xu DD, Ungvari GS, Ng CH, Chow IHI, Xiang YT (2018). Prevalence of suicidal behaviors in patients with major depressive disorder in China: A comprehensive meta-analysis. J Affect Disord.

[31] Novick DM, Swartz HA, Frank E (2010). Suicide attempts in bipolar I and bipolar II disorder: a review and meta-analysis of the evidence. Bipolar Disord.

